# Increase in potato yield by the combined application of biochar and organic fertilizer: key role of rhizosphere microbial diversity

**DOI:** 10.3389/fpls.2024.1389864

**Published:** 2024-05-15

**Authors:** Jianwei Hou, CunFang Xing, Jun Zhang, Zuhua Wang, Min Liu, Yu Duan, Hui Zhao

**Affiliations:** ^1^ Institute of Resources, Environment and Sustainable Development, Inner Mongolia Academy of Agricultural and Animal Husbandry Sciences, Hohhot, Inner Mongolia, China; ^2^ School of Agroforestry Engineering and Planning, Tongren University, Tongren, Guizhou, China

**Keywords:** biochar, organic fertilizer, equal carbon ratio, chemical properties, rhizosphere microorganism, potato yield

## Abstract

**Purpose:**

The large-scale planting of potatoes leads to soil degradation, thus limiting the potato yield. An effective method of improving soil quality involves the combined application of biochar and organic fertilizer. However, the proportion of biochar and organic fertilizer at which potato yield can be improved, as well as the improvement mechanism, remain unclear.

**Methods:**

A combined application experiment involving biochar (B) and organic fertilizer (O) with four concentration gradients was conducted using the equal carbon ratio method. On this basis, rhizosphere soil fertility, bacterial community composition, and bacterial diversity in potato crops, as well as the potato yield difference under different combined application ratios, were investigated. Then, the direct and indirect effects of these factors on potato yield were analyzed.

**Results:**

The results suggest that soil fertility was improved by the combined application of biochar and organic fertilizer, with the best effect being achieved at a ratio of B:O=1:2. The dominant bacterial communities in the potato rhizosphere included *Proteobacteria*, *Actinobacteria*, *Gemmatimonadetes*, *Chloroflexi*, and *Bacteroidetes*. When compared to the control, the relative abundance and diversity index of soil bacteria were significantly improved by the treatment at B:O=1:2, which exerted a stronger effect on improving the relative abundance of beneficial bacteria. Soil available phosphorus (AP), soil pH (SpH), and soil organic carbon (SOC) explained 47.52% of the variation in bacterial composition. Among them, the main factor was the content of soil available nutrients, while SpH generated the weakest effect. The bacterial diversity index showed a significant positive correlation with soil AP, SOC, available potassium (AK), total nitrogen (TN), and C/N ratio, and a significant negative correlation with SpH. Bacterial diversity directly affected the potato yield, while soil fertility indirectly affected potato yield by influencing the soil bacterial diversity.

**Conclusion:**

The combined application of biochar and organic fertilizer elevates potato yield mainly by improving the diversity of bacterial communities in potato rhizosphere soil, especially the combined application of biochar and organic fertilizer at a 1:2 ratio (biochar 0.66 t ha^-1^+organic fertilizer 4.46 t ha^-1^), which made the largest contribution to increasing potato yield.

## Graphical Abstract

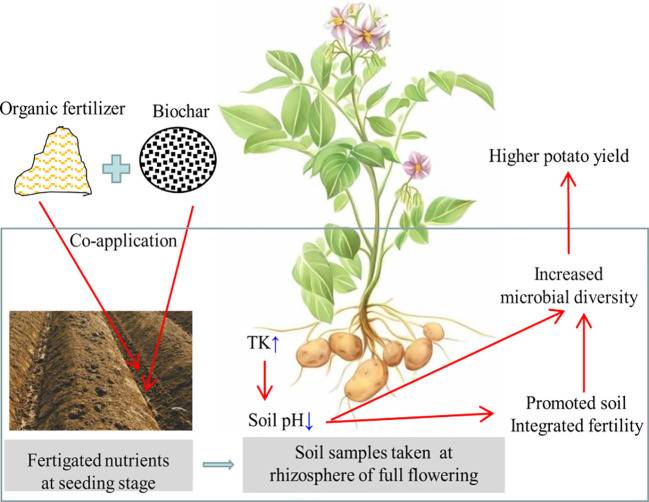

## 1 Introduction

As one of the main agricultural crops in China, potato serves key roles in maintaining national life and economic development. However, with the continuous expansion of the potato planting area in recent years, soil problems have become increasingly prominent in potato planting areas, reducing the potato yield (Fidel et al., 2017)and thus making the alleviation or elimination of soil depletion in potato producing areas an urgent issue (Wang et al., 2016; Zhang et al., 2017). An important measure that can be used to rapidly cultivate hungry soil is the application of organic fertilizer (Chen et al., 2015). In particular, soil carbon pool reserves and nutrient content can be comprehensively improved by the combined application of biochar and organic fertilizer to further improve the nutrient absorption efficiency of crops and increase their yield (Li et al., 2015). However, the soil improvement effect of the combined application of organic fertilizer and biochar can be restricted by many factors, such as the type of organic fertilizer used (Zhao et al., 2015), the preparation materials and processes of biochar (Zhang et al., 2015), and the soil type (Zhang et al., 2013), thus making the popularization and application of this improvement measure in production practice challenging. Therefore, it is vitally important to seek a combined application method of biochar and organic fertilizer that can be easily promoted for crop production.

Biochar is a type of highly aromatic carbon-rich product formed by the pyrolysis of biomass materials such as crops under limited oxygen or anoxic conditions (Novak et al., 2019), whereas organic fertilizer is a type of carbon-containing material that is rich in plant nutrients, with most nutrients in the two types of fertilizers binding to organic carbon (Pyakurel et al., 2019). Therefore, based on the carbon content in organic materials, fertilizers such as biochar and organic fertilizers can be applied at a specific mix proportion according to the carbon input, which can be easily popularized and applied given different sources of biochar and organic fertilizers, as well as the varying basic fertility levels of farmlands. Specifically, the assumed equal carbon input involves calculating the total carbon input (t·ha^-1^) in the study area according to the recommended dosage (t·ha^-1^) of biochar and the organic carbon content (%) of potatoes, thus setting a proportional gradient (i.e., the mass ratio of organic carbon) with the total carbon input as a fixed value and finally converting it into a field dosage of biochar and organic fertilizer. Therefore, this method will help popularize the combined application of biochar and organic fertilizer. However, the influence of biochar and organic fertilizer with equal carbon inputs on soil fertility has been scarcely investigated, and the most suitable mix proportion for soil cultivation remains unclear.

In addition to abiotic factors such as soil fertility, soil microorganisms also serve as influencing factors for crop yield. For instance, Wang et al. (1991) found that soybean yield can be increased by maintaining the diversity and activity of soil microorganisms. Additionally, Wu et al (Wu and Wang, 2006). highlighted that soils subjected to continuous cropping will exhibit a decline in the diversity index, abundance, and evenness of microbial communities, resulting in a significant reduction in cucumber yield. Moreover, a study conducted by Zhang et al. (2012) proved that corn yield and biological yield per plant presented significant or extremely significant positive correlations with soil microbial quantity, noting that reasonable fertilization can increase the microbial quantity and enzymatic activity in the soil, thus effectively improving soil fertility and crop growth and nutrition while achieving the purpose of increasing crop yield. Therefore, soil microbial diversity is of immense importance to the functions of soil ecosystems and the crop field, with high microbial diversity having the ability to enhance the stability of microbial functions and promote increased crop yield (Deng et al., 2016). However, the proportion of biochar and organic fertilizer with equal carbon inputs at which the soil microbial diversity, soil fertility, and potato yield can be optimally improved remains unclear, as does the influencing mechanism of this fertilization mode on potato yield.

Guizhou is a major potato-producing province and one of the earliest potato cultivation areas in China, with both the potato planting area and yield ranking second at the national level. However, the average yield per unit area in Guizhou is not high, ranking approximately 16^th^ in the country. In Guizhou, low soil fertility is an existing problem that limits potato yield (Liu, 2005). Therefore, quantitatively investigating specific fertilization modes and proposing the application of biochar and organic fertilizer strategies that can contribute to high potato yield represents an urgent need. In response to this need, a gradient experiment involving four mix proportions of biochar and organic fertilizer was performed in the yellow soil of a dry land plot to assess potato yield, soil fertility, and soil microbial characteristics, with the primary aim of solving the following issues: 1) determining the influence of various mix proportions of biochar and organic fertilizer with equal carbon inputs on soil fertility; 2) determining the influence of the various mix proportions of biochar and organic fertilizer on bacterial community diversity in the potato rhizosphere; 3) determining the influencing mechanism of the combined application of biochar and organic fertilizer on potato yield. By solving the aforementioned issues, potato yield could be increased and key technologies could be promoted.

## 2 Materials and methods

### 2.1 Overview of the experimental area

The experimental area was set in Tangjiazhai, Chuandong Street, Bijiang District, Tongren City, Guizhou Province (27°47′32″ N,109°13′8″ E). The experimental area is located in the northeast of Guizhou Province and to the east of Tongren City. The area has a humid subtropical monsoon climate, with an average annual temperature of 16.9°C, a frost-free period of 291 days, annual rainfall of 1,265.4 mm, an annual sunshine duration of 1,171 h, and an elevation of 434 m. The soil is mountainous yellow soil with a silty loam texture.

### 2.2 Experimental design

In this experiment, a combined application experiment involving biochar (B) and organic fertilizer (O) with four concentration gradients was conducted using the equal carbon ratio (i.e., mass ratios of organic carbon) method, namely B:O=1:0, B:O=1:1, B:O=1:2, and B:O=1:3. Additionally, a blank control treatment (CK) without the application of biochar or organic fertilizer was also included, thus totaling five treatments (**Table 1**). A randomized experimental design was adopted, and the experiment was repeated three times, with a plot area of 30 m^2^. The tested potato variety was “Nongyin No. 2”. The tested biochar was corn straw biochar whose pH, *ω*(*C*), *ω*(*N*), *ω*(*P*), *ω*(*K*), specific surface area, total pore volume, and average pore size were 10.16, 52%, 11.2 g·kg^-1^, 4.03 g·kg^-1^, 14.6 g·kg^-1^, 158.7 m^2^·g^-1^, 0.33 mL·g^-1^, and 2.42 nm, respectively. The organic fertilizer was sheep manure (*ω*(*C*) =15.7%, *ω*(*N*)=0.75%, *ω*(*P*)=0.51%, and *ω*(*K*)= 0.42 g·kg^-1^, respectively).

**Table 1:** Equal carbon input design of biochar and organic fertilizer.

Treatment	Amount of organic material application (t·ha^-1^)				Total carbon input (t·ha^-1^)
	Biochar	Organic carbon input	Sheep manure (dry)	Organic carbon input	
CK	0	--	0	--	--
B:O=1:0	2.00	1.04	0	0	1.04
B:O=1:1	1.00	0.52	3.31	0.52	
B:O=1:2	0.66	0.35	4.46	0.70	
B:O=1:3	0.50	0.26	4.97	0.78	

CK: no application of biochar or organic fertilizer; B:O=1:0: application of biochar without organic fertilizer; B:O=1:1: combined application of biochar and organic fertilizer at a 1:1 ratio; B:O=1:2: combined application of biochar and organic fertilizer at a 1:2 ratio; B:O=1:3: combined application of biochar and organic fertilizer at a 1:3 ratio; “-”: not measured.

Seeds were sown by hand on 25 February 2023 in furrows about 15 cm deep, the plant spacing was 30 × 50 cm, seeding approximately 75000 plants·ha^−2^. Biochar and organic fertilizers were applied to the ridge at one time when the potato was sown. In which all P and K fertilizers were used as the basal fertilizer, 50% N fertilizer as the basal fertilizer, and 50% N fertilizer as the topdressing. The remaining 50 per cent of the nitrogen fertilizer is spread in four equal portions around the roots of potato plants on 15 March, 15 April, 15 May, and 15 June each year, after which the soil was turned over and covered. The proposed dosage of biochar and chemical fertilizer was 2 t·ha^-1^ (Luo, 2021), N 150 kg·ha^-1^, P_2_O_5_ 135 kg·ha^-1^, and K_2_O 135 kg·ha^-1^ (Zhang et al., 2017), respectively. The nitrogen fertilizer used in the experiment was urea (N 460 g·kg^-1^). The phosphate fertilizer was superphosphate (P_2_O_5_ 460 g·kg^-1^). The potassium fertilizer was potassium chloride (K_2_O 600 g·kg^-1^).

### 2.3 Sample collection

At the full flowering stage of potatoes (April 15th), the side rows in each plot were removed. Then, five potato plants with the same growth vigor were determined by an “S”-shaped point arrangement. Firstly, we cleaned up the weeds and dead leaves around the potatoes; then, we uprooted the potato plants, and after shaking off the larger pieces of soil from the root system, we collected small pieces of soil or soil particles attached to the root surface (about 2-3 mm) as rhizosphere soil samples (Lu et al., 2015), soil samples in the same plot were evenly mixed as a mixed sample, and a total of 15 rhizosphere samples were collected. Some of the mixed soil samples were air-dried and stored to determine soil physiochemical properties, while others were stored in a refrigerator at -80°C to extract the total deoxyribonucleic acid (DNA) of microorganisms for high-throughput sequencing.

In addition, five whole plants collected in each plot were tested on 30 June for seeds, and potato tubers were weighed. The number of missing plants and variant plants in the plot were investigated. The actual final harvest plants in the plot were determined; the entire harvest was taken as the yield.

### 2.4 High-throughput sequencing of soil bacteria

The high-throughput sequencing of soil bacteria was conducted by Shanghai Personal Biotechnology Co., Ltd., which included genomic DNA extraction, polymerase chain reaction (PCR) amplification, sequencing, sequence processing, and analysis, as follows:

(1) DNA extraction: Soil DNA was extracted using a DNA extraction kit (OMEGA M5635-02, USA). Then, the DNA concentration was detected via agarose gel electrophoresis and NanoDrop2000.
(2) PCR amplification: With genomic DNA as a template, the V3-V4 region of bacterial 16S ribosomal ribonucleic acid (rRNA) genes were amplified using the primers 343F (5’-TACGGRAGGCAGCAG-3’) and 798R (5’-AGGGTATCTAATCCT-3’) (Tks Gflex DNA Polymerase, Takara).
(3) Sequencing: The PCR products were purified, quantified, and homogenized based on the 16S rDNA sequence to construct libraries whose quality levels were inspected on LabChip. Then, the qualified library was subjected to high-throughput sequencing using Illumina MiSed.
(4) Sequence processing and analysis: The original sequence was subjected to primer removal, quality filtering, denoising, splicing, and chimera removal using the DADA2 method to construct a table of amplicon sequence variants (ASVs). Firstly, DADA2 was deduplicated to obtain the representative sequences of ASVs. Then, different types of sequences were identified by using the Silva database (Release 132, http://www.Arb-silva.De) as a template sequence for the identification of ASV taxonomic status.

### 2.5 Soil physiochemical properties

Soil pH (SpH) was determined at a 1:5 (w/v) soil/distilled water ratio using a pH meter (Mettler Toledo Delta 320 (Zhai et al., 2015)). Soil organic carbon (SOC) was determined using the potassium dichromate oxidation method (Partey et al., 2014). Soil available N (AN) was measured using the alkaline hydrolysis diffusion method (Wang et al., 2018). Soil available P (AP) and total P (TP) were detected by Mo–Sb colorimetry using an EdgeLight UV754 spectrophotometer (Shanghai, China (Zhai et al., 2015)). Soil available K (AK) and total K (TK) were analyzed by ICP–OES (PerkinElmer, Waltham, USA (Yu et al., 2016)). Soil total N (TN) was determined by a Kjeldahl nitrogen analyzer (KDN-812, Shanghai, China) after digestion with concentrated sulphuric acid (Zhai et al., 2015). Soil C/N is the ratio of SOC to TN.

### 2.6 Statistical analysis of data

First, the soil integrated fertility index (IFI) was calculated using the Nemerow integrated index method (Yang et al., 2015), and the soil microbial diversity index was determined. These measures, as well as the influences of fertilization at different mix proportions on soil IFI, soil physiochemical properties, soil microbial diversity, and potato yield, were analyzed via one-way analysis of variance (ANOVA) using SPSS 27.0, Tukey HSD *post hoc* multiple comparisons when ANOVA results showed differences.

MetagenomeSeq analysis was performed using the R (V.4.2.1) software package vegan to discriminate the significantly up-regulated phylum and genus. Moreover, the bacterial Bray-Curtis dissimilarity index between different mix proportions of fertilizers was distinguished using Kruskal-Wallis tests and Wilcoxon signed-rank tests.

The indirect and direct influencing paths of fertilization on potato yield were analyzed using a structural equation. Since the soil fertility, microbial yield, and potato yield were increased by all combined fertilizer application schemes, a structural equation was constructed using all processing data. As revealed by the results of previous studies, soil physiochemical properties, fertility, and soil microorganisms have direct effects on potato yield, while soil fertility and physiochemical properties can affect potato yield by acting on the soil microbial diversity. Therefore, a structural equation regarding the influence of biodiversity on potato yield was established according to this conceptual model. The model fitting effect was discriminated by using Fisher’s C and *p*>0.05. At the beginning of the model, unimportant variables with calculated correlation coefficients greater than 0.7 were excluded. Finally, TP, TK, SpH, IFI, and soil microbial Shannon index were included in the model. The structural equation was established using the piecewiseSEM package in R (V.4.2.1).

In addition, indices of alpha diversity were calculated using the vegan package in the R language software, community richness was estimated using the Pielou index and Richness index to estimate the number of ASVs in the soil bacterial community, and community evenness was calculated using the Shannon and Simpson indices to calculate the diversity of the soil bacterial community.

## 3 Results

### 3.1 Effect of the proportion of biochar and organic fertilizer on potato yield and soil IFI

Potato yield was significantly increased by the combined application of biochar and organic fertilizer (*p*<0.05), and the largest potato yield was achieved in the B:O=1:2 treatment, which was 1.48 times higher than that obtained in the CK treatment. The potato yields obtained in the B:O=1:3, B:O=1:1, and B:O=1:0 treatments followed successively, being 1.39 times, 1.28 times, and 1.16 times higher than that obtained in the CK treatment, respectively (**Table 2**).

The mix proportion of biochar and organic fertilizer significantly affected the basic chemical properties of soil (**Table 2**). Compared to the CK treatment, the combined application of biochar and organic fertilizer reduced SpH by an average of 0.4 pH units (*p*<0.05), among which the B:O=1:0 treatment reached the lowest SpH but was accompanied by the highest TK and C/N. The highest AK, TP, and SOC were achieved in the B:O=1:1 treatment. The highest AP was obtained in the B:O=1:2 treatment. The highest TN was achieved in the B:O=1:3 treatment, with an increase of 64.0% when compared with that in the CK treatment, and it increased with increasing proportions of organic fertilizer (*p*<0.05). AN, TK, and TP were non-significantly influenced by the mix proportion of biochar and organic fertilizer (**Table 2**). According to the soil fertility classification based on IFI, the IFI of the CK treatment was 0.65<0.90, which falls under fertility level IV (a barren fertility level). The IFI of other treatments varied within the range of 1.21–1.26, corresponding to fertility level III (a general fertility level).

**Table 2:** Rhizosphere soil properties at different mix proportions of biochar and organic fertilizer.

Index	CK	B:O=1:0	B:O=1:1	B:O=1:2	B:O=1:3
Potato Yield (t·ha^-1^)	12.70 ± 0.50d	14.77 ± 0.10c	16.31 ± 0.97bc	18.79 ± 0.39a	17.61 ± 0.42ab
pH	7.35 ± 0.06a	6.88 ± 0.10c	7.00 ± 0.02bc	7.07 ± 0.02b	7.00 ± 002bc
AN(mg·kg^-1^)	10.50 ± 0.11a	10.50 ± 0.21a	10.50 ± 0.00a	10.50 ± 0.25a	10.50 ± 0.86a
AP(mg·kg^-1^)	7.53 ± 0.28c	19.02 ± 0.10c	49.15 ± 4.75b	72.34 ± 6.48a	64.04 ± 2.83a
AK(mg·kg^-1^)	42 ± 4c	453 ± 31b	692 ± 74a	686 ± 71a	724 ± 71a
TK(g·kg^-1^)	10.32 ± 1.88a	13.43 ± 0.77a	12.51 ± 0.67a	12.43 ± 0.61a	11.21 ± 0.02a
TP(g·kg^-1^)	0.08 ± 0.03a	0.11 ± 0.02a	0.12 ± 0.01a	0.10 ± 0.02a	0.09 ± 0.02a
SOC(g·kg^-1^)	4.41 ± 1.05b	24.8 ± 0.85a	27.04 ± 2.56a	25.5 ± 0.94a	25.7 ± 1.56a
TN(g·kg^-1^)	0.67 ± 0.22e	1.18 ± 0.15d	1.45 ± 0.04c	1.70 ± 0.08b	1.86 ± 0.01a
C/N	6.20 ± 8.01d	21.09 ± 1.54a	18.67 ± 1.94ab	14.98 ± 1.25bb	13.84 ± 0.48c
IFI	0.65 ± 0.04b	1.21 ± 0.02a	1.24 ± 0.01a	1.26 ± 0.01a	1.25 ± 0.01a

The numerical value (mean ± standard deviation) indicates the absolute quantity of each type of soil property. B, biochar; O, organic fertilizer; AN, available N; AP, available P; AK, available K; TN, total N; TP, total P; TK, total K; SOC, soil organic carbon; C/N, the ratio of SOC to TN; IFI, integrated fertility index. Different lowercase letters in the same row indicate significant differences between treatments (p<0.05).

### 3.2 Effects of the mix proportion of biochar and organic fertilizer on soil microbial composition and diversity

A total of 11,488 ASVs were detected in all soil samples, belonging to 24 phyla, 75 classes, 207 orders, 352 families, and 632 genera (excluding unidentified fungi). *Proteobacteria*, *Actinobacteria*, *Gemmatimonadetes*, *Chloroflexi*, and *Bacteroidetes* were the dominant bacteria in the soil, and the sum of their average relative abundance was greater than 91% (**Supplementary Figures S1A, B**). With the CK treatment as the control group and the B:O=1:0, B:O=1:1, B:O=1:2, and B:O=1:3 treatments as upregulation groups, the MetagenomeSeq analysis results suggest that the relative abundance of core bacteria was significantly elevated by the combined application of biochar and organic fertilizer (**Supplementary Figures S2A–D**). The effect of the B:O=1:2 treatment (**Supplementary Figure S2B**) was more apparent, with the significantly upregulated genera of Subgroup_6, *Glycomyces*, *Promicromonospora*, *Streptomyces*, *Gemmatimonas*, S0134_terrestrial_group, *Dongia*, *Mesorhizobium*, *Steroidobacter*, and *Lysobacte*, which mainly belonged to three dominant bacterial phyla (*Actinobacteria*, *Gemmatimonadetes*, and *Proteobacteria*).

**Figure 1:**
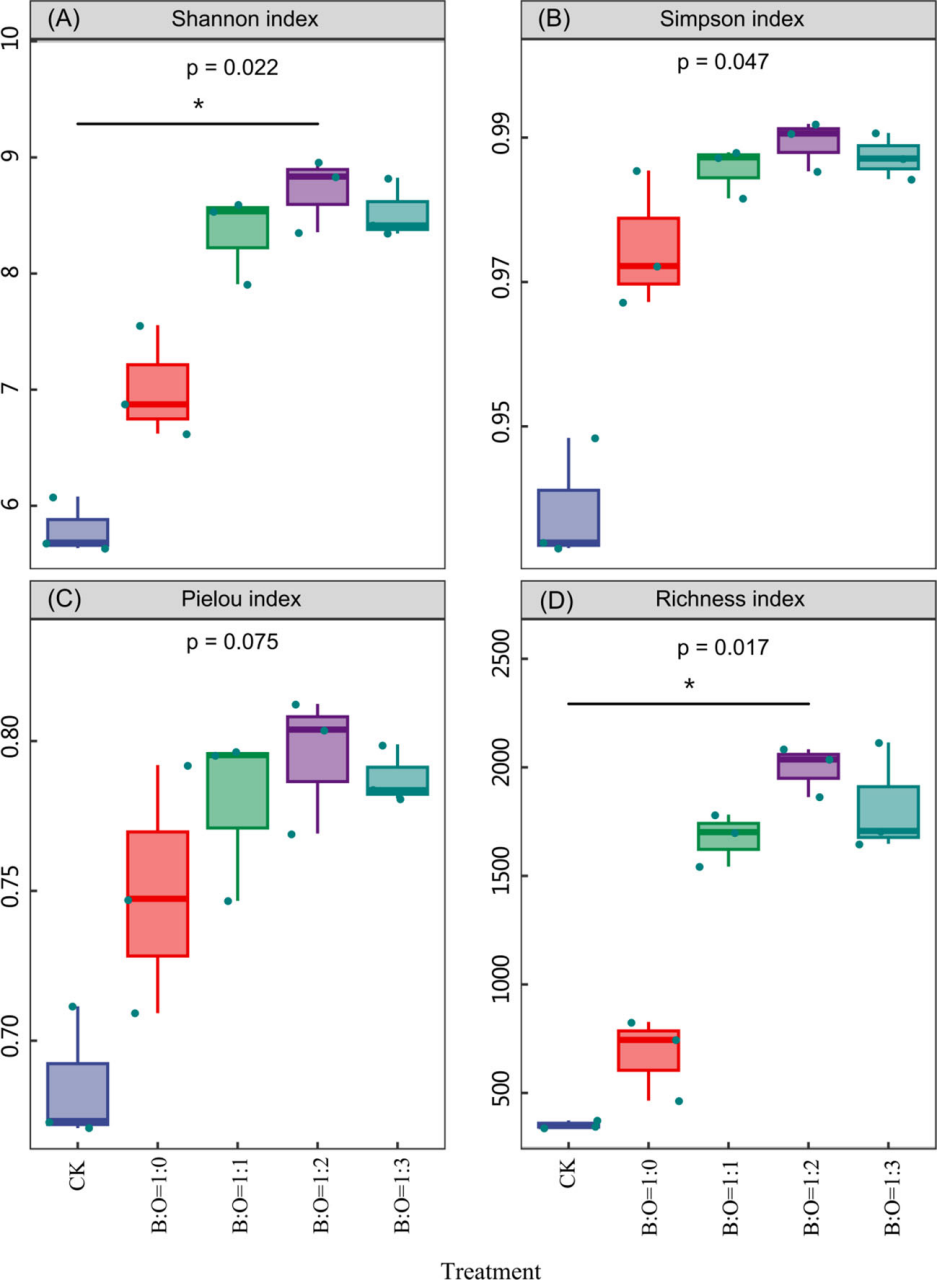
Shannon index **(A)**, Simpson index **(B)**, Pieiou index **(C)**, and richness index **(D)** of potato rhizosphere bacteria under different proportions of biochar and organic fertilizer. The number below the diversity index label is the *p*-value of Kdunn’s backtesting, *p<0.05.

The diversity analysis results revealed that the combined application of biochar and organic fertilizer could significantly affect the Shannon index, Simpson index, Pielou’s index, and richness index of bacteria, all of which reached their highest values in the B:O=1:2 treatment and their lowest values in the CK treatment (**Figures 1A–D**). According to the backtesting analysis of Kdunn’s test, significant differences were observed in the Shannon index and richness index among four groups—CK, B:O=1:0, B:O=1:1, and B:O=1:2 (*p*<0.05) (**Figures 1A, D**)—however, no significant differences were observed in the Simpson index or Pielou’s index (**Figures 1B, C**). According to the hierarchical clustering analysis, all samples could be divided into three classes: (I) B:O=1:1, B:O=1:2, and B:O=1:3 were clustered into one class; (II) B:O=1:0 formed an independent class; (III) CK was an independent class (**Supplementary Figure S3A**). This was determined based on the intergroup difference analysis using the Bray-Curtis algorithm, and significant differences were observed among the three classes in the Bray-Curtis dissimilarity index (*p*<0.05). The dissimilarity index of Class (I) was the highest, followed by those of Classes (II) and (III), successively (**Supplementary Figure S3B**), thus indicating that the bacterial community composition exhibits the highest similarity among samples in the B:O=1:1, B:O=1:2, and B:O=1:3 treatments, followed by those in the B:O=1:0 treatment and in CK successively. This result was also proven by the hierarchical clustering analysis (**Supplementary Figure S3A**).

**Figure 1**. Rhizosphere bacterial *α* diversity index at different mix proportions of biochar and organic fertilizer. The number below the diversity index label is the *p*-value of Kdunn’s backtesting, *p<0.05.

### 3.3 Soil environmental factors affecting bacterial community composition and diversity

After the nine explanatory variables—including SpH and C/N—were forwardly selected, the remaining six variables were SpH, AP, TN, AK, SOC, and TP. Then, a redundancy analysis (RDA) model (**Supplementary Figure S4A**) was constructed with the bacterial communities. The results revealed that SpH, AP, and SOC were significant factors influencing bacterial communities (*p*<0.05). The influences of SpH, soil total nutrient content (TN, SOC, and TP), and soil available nutrient content (AP and AK) on bacterial communities were further analyzed by a variance decomposition analysis (**Supplementary Figure S4B**). It was uncovered that soil available nutrient content (50.5%) was the primary factor affecting bacterial communities, followed by soil total nutrient content (46.9%), and SpH (12.4%), which exerted the minimum effect.

The correlation analysis results showed that soil bacterial diversity was significantly influenced by changes in soil physiochemical factors (**Supplementary Figure S5**). In addition to soil AN, TK, and TP, other soil environmental factors significantly influenced bacterial diversity. Among them, soil AP, AK, SOC, and TN exerted the greatest influence, showing significantly or extremely significantly positive correlations with bacterial diversity, followed by C/N and pH, which presented significantly positive and negative correlations with bacterial diversity, respectively.

### 3.4 Potential effects of soil fertility and bacterial diversity on potato yield

**Figure 2:**
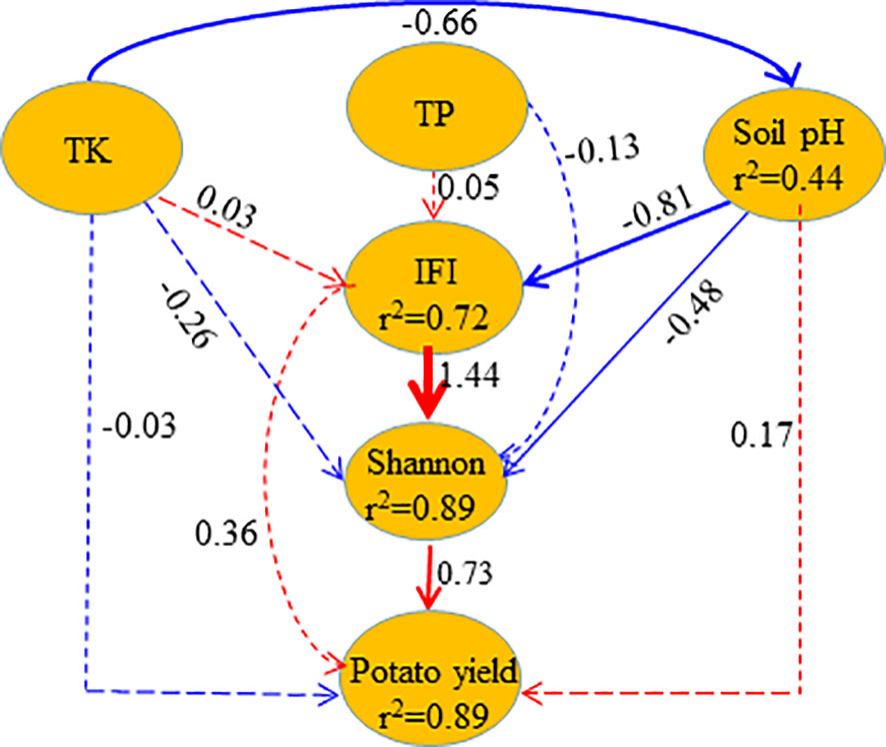
Structural equation model for the influence of soil fertility and soil microbial diversity on potato yield. Red and black lines represent positive and negative influences, respectively. Solid and dotted lines represent significant and insignificant levels, respectively. Line thickness represents the size of the path coefficient (Fisher’s *C*=0.144; *p*=0.998; *df*=4). .

The structural equation model reflected that soil microbial diversity directly affected potato yield, while soil characteristics only indirectly influenced potato yield (**Figure 2**). Moreover, TK could increase potato yield through two indirect paths (path coefficient: 0.79). The first path involves TK increasing microbial diversity by lowering the SpH and improving soil fertility, thereby elevating potato yield (path coefficient: 0.56). The second path involves TK increasing microbial diversity by lowering the SpH, thereby elevating potato yield (path coefficient: 0.23). Overall, 89% of the potato yield could be explained by soil characteristics and soil microorganisms, 44% of SpH changes could be attributed to soil TK, 72% of soil fertility changes could be explained by SpH, and 89% of soil microbial diversity could be explained by soil fertility.

## 4 Discussion

Ameloot et al. (2013) found that biochar can stimulate changes in soil bacterial community diversity, which was also verified by the sole application of biochar (B:O=1:0) in this study (**Figures 1A–D**). Notably, the combined application of biochar and organic fertilizer was better at improving rhizosphere bacterial diversity and abundance than the sole application of biochar, especially the B:O=1:2 treatment (**Figures 1A–D**). Despite its ability to provide a good habitat for microorganisms (Lehmann et al., 2011), biochar is mostly inert coal instead of a microbial active carbon source; however, this deficiency can be rightly compensated by organic fertilizer. If applied in a combined fashion, the two can facilitate the proliferation of certain specific bacterial communities and enhance their abundance (Taschen et al., 2017). Additionally, a biochar and organic fertilizer mix proportion of 1:2 may be the most suitable for this purpose. Some studies have noted that the combined application of biochar and organic fertilizers affects soil microbial community structure because it can change the physiochemical properties of microbial colonization habitat (Prayogo et al., 2014). As such, particular emphasis should be placed on the correlations of the change in soil microbial communities with the changes in soil nutrient turnover and utilization in follow-up studies. Even so, this study supports the notion that the combined application of biochar and organic fertilizer can significantly change the bacterial community composition (**Supplementary Figure S1**) and enhance bacterial diversity (**Figure 1**), which is strongly correlated with a neutral pH value and high nutrient content in fertilized soil samples (**Supplementary Figures S4A, B**; **S5**). Interestingly, both the soil bacterial abundance and diversity were highest in the B:O=1:2 treatment; however, the soil nutrient content was not the maximum. This result seemed to support the hypothesis of Nannipieri et al. (1983), which noted that each type of soil has a specific biological content under equilibrium conditions. In other words, no obvious microbial changes will occur when soil conditions are continuously optimized on the precondition that relatively suitable conditions are provided for the growth of soil microbial communities by the soil microenvironment.

Importantly, the application of organic fertilizer improves the utilization efficiency of agricultural wastes, which can popularize the combined application technology of organic and inorganic fertilizers (Zhang et al., 2017). Within the study area, the basic soil fertility level was improved after the combined application of biochar and organic fertilizer (**Table 2**). In particular, soil AK, SOC, and TN were elevated from the original Level 5 to Levels 1, 1, and 2–3, respectively (**Table 2**). To reflect the fact that the higher crop requirements for soil properties might not always lead to better results, the soil fertility level was comprehensively evaluated using the Nemerow integrated index method (Yang et al., 2015), and the IFI of CK was found to be 0.65<0.90, belonging to the barren fertility level. The B:O=1:0, B:O=1:1, B:O=1:2, and B:O=1:3 treatments reached an IFI value of 1.21–1.26 (reaching a general fertility level), among which the IFI value was greatest in the B:O=1:2 treatment (**Table 2**). This suggests that the combined application of biochar and organic fertilizer mainly increases soil AK, SOC, and TN, thus enhancing the soil IFI. Nevertheless, the study area had an extreme shortage of soil AN, TP, and AP, and attention should be paid to the timely supplementary application of nitrogen and phosphorus fertilizers in production, which is mainly related to the input of organic fertilizers in fore crops, frequent rainfall, serious nitrogen leaching loss, and AP fixation by yellow soil in the study area (Chen et al., 2006).

Previous studies have revealed that the combined application of biochar and organic fertilizers can improve soil carbon pool reserves and nutrient content more comprehensively while also increasing crop yield (Doan et al., 2015), especially in low-fertility soil and degraded land (Minhas et al., 2020). For example, the combined application of biochar and organic fertilizers improves the yield and quality of *Hylocereus polyrhizus* by improving soil properties, with 3% biochar+45 t ha^-1^ organic fertilizer being the recommended combination leading to the best synergistic effect (Chen et al., 2022). Other studies have shown that the combined application of biochar and organic fertilizers can enhance the productivity of plants by improving SpH and fertility (Ye et al., 2019; Ye et al., 2020). In this study, it was also proven that soil fertility and crop yield could be improved by the combined application of biochar and organic fertilizer (**Table 2**). Moreover, a structural equation model was established in this study to reveal the direct and indirect effects of soil fertility and microbial diversity on potato yield. The model results indicate that the potato yield was directly and positively affected by bacterial diversity, while soil fertility indirectly affected potato yield by influencing soil bacterial diversity (**Figure 2**), which is inconsistent with the research of Sun (2022) and Xiao et al. (2021). They believed that soil TN and organic matter (carbon) have a direct positive impact on potato (rice) yield, while bacterial diversity has no significant impact on the yields of these crops. This inconsistency could be attributed to the characteristics of biochar and organic fertilizers. Firstly, biochar primarily contains inert carbon, which does not easily react with the soil; however, its porous structure can provide a suitable habitat for soil microorganism growth (Mishra et al., 2021) and the nutrient cycles of microbial communities (Gao and DeLuca, 2018). Secondly, although organic fertilizers cannot be used as long-term and sufficient nutrient sources for plants, their rich activated carbon content can provide sufficient energy for soil microorganisms (Shi et al., 2020), which can enhance soil microbial reproduction, soil properties, and bioactivity (Uzoma et al., 2011). Therefore, the combined application of biochar and organic fertilizer can overcome their respective disadvantages, more comprehensively improve soil carbon pool reserves and nutrient content, and thus improve rhizosphere microbial reproduction and diversity. Additionally, the transformation of community functions may be facilitated by changes in the bacterial community composition induced by the combined application of biochar and organic fertilizers. In this study, the relative abundance of such beneficial bacteria as *Streptomyces*, *Gemmatimonas*, *Lysobacter*, *Mesorhizobium*, and *Steroidobacter* was significantly upregulated by the combined application of biochar and organic fertilizer at 1:2 (**Supplementary Figure S2B**). Based on the physiological functions of beneficial bacteria, *Streptomyces* can produce antibiotics and plant growth hormone substances, promote plant growth, and improve plant resistance to biotic and abiotic stresses (Kim et al., 2011). *Gemmatimonas* can relieve the harmful effects of nitrites and inhibit pathogenic bacteria (Zhang et al., 2017). *Lysobacter*, a new-type biocontrol bacterium with biocontrol potential, exerts a strong bacteriolytic and antibacterial effect accompanied by strong antagonistic activity against a variety of plant pathogenic fungi, bacteria, and nematodes (Liu, 2016). *Mesorhizobium* can effectively control soil-borne plant and fungal diseases, with a field preventive effect on the bacterial wilts (ginger blast) of potato, tomato, eggplant, and pepper reaching over 70%, indicating a highly significant yield-increasing effect (Marta et al., 2014). It can be seen that increases in beneficial bacterial communities such as *Actinobacteria*, *Gemmatimonadetes*, *Chloroflexi*, and *Bacteroidetes* can not only promote plant growth and resist diseases but also promote higher crop yields, which corroborates the highest rhizosphere biodiversity (**Figures 1A–D**) and potato yield (**Table 1**) achieved in the B:O=1:2 treatment.

Notably, since *Gemmatimonas* is an autotrophic denitrifying microorganism (Zhang et al., 2020), an increase in its abundance will likely increase the probability of nitrogen anti-digestion and trigger nitrogen loss in addition to relieving the harm of nitrites and inhibiting pathogenic bacteria. On this basis, the elevated abundance of *Gemmatimonas* in the potato rhizosphere due to the combined application of biochar and organic fertilizer at 1:2 (**Supplementary Figure S2B**) might be detrimental to nitrogen fixation in soil. However, according to some studies, biochar and organic fertilizers, which show very strong absorption capacity, can retain soil nutrients (Wang, 2022). For instance, the combined application of biochar and organic fertilizers significantly enhances the adsorption and immobilization effect of NH_4_^+^-N and NO_3_^–^N in the soil of paddy fields and reduces the nitrogen loss, thus increasing the nitrogen use efficiency (Warnock et al., 2007). As the coating material of urea, biochar can reduce ammonia volatilization loss by 16.7–31.8% when compared to ordinary urea (Ji et al., 2008). Therefore, the retention effect of biochar combined with an organic fertilizer at a 1:2 ratio on nutrients may be greater than the negative impact caused by the increase of *Gemmatimonas* abundance, which should be studied further.

## 5 Conclusion

Biochar with organic manure is an effective fertilisation strategy to enhance the fertility level of lean yellow soil, especially the B:O=1:2 treatment has the best soil fertilization effect, but soil AN, TP, and AP remained extremely low in the study area under this fertilisation strategy.

Soil AP, SpH, and SOC significantly influenced bacterial communities, while soil AP, AK, SOC, TN, C/N, and pH were significantly correlated with bacterial community diversity, Biochar with organic fertiliser especially B:O=1:2 treatment significantly increased the level of bacterial diversity.

Bacterial diversity has a direct effect on potato yield, and it plays a key role in enhancing potato yield with biochar and organic fertiliser. Soil fertility index, on the other hand, can indirectly affect potato yield by influencing bacterial diversity and is another important factor affecting potato yield in the study area.

Considering the soil cultivation effect, rhizosphere bacterial community diversity, and potato yield, B:O=1:2 is the recommended combination for obtaining the optimal synergistic effect (i.e., biochar 0.66 t ha^-1^ + organic fertilizer 4.46 t ha^-1^). Meanwhile, nitrogen and phosphorus fertilizers should be appropriately applied as a supplement to meet the needs of potato growth.

## Data availability statement

The original contributions presented in the study are included in the article/**Supplementary Material**. Further inquiries can be directed to the corresponding author.

## Author contributions

HJ: Conceptualization, Data curation, Methodology, Validation, Visualization, Writing – original draft, Writing – review & editing. XC: Resources, Writing – review & editing. ZJ: Formal analysis, Funding acquisition, Investigation, Project administration, Writing – review & editing. WZ: Data curation, Software, Writing – review & editing. LM: Software, Writing – review & editing. YD: Funding acquisition, Visualization, Writing – original draft. ZH: Supervision, Writing – original draft.

## References

[B1] Ameloot N. Graber E. R. Verheijen F. G. Neve S. D. (2013). Interactions between biochar stability and soil organisms: review and research needs. Eur. J. Soil Sci. 64, 379–390. doi: 10.1111/ejss.12064

[B2] Chen L. Li X. Peng Y. Xiang P. Zhou Y. Yao B.. (2022). Co-application of biochar and organic fertilizer promotes the yield and quality of red pitaya (Hylocereus polyrhizus) by improving soil properties. Chemosphere 294, 133619. doi: 10.1016/j.chemosphere.2022.133619 35041821

[B3] Chen L. J. Sun B. Jin C. Jiang Y. J. Chen L. (2015). Effect of organic manure and biochar with equal amount of carbon input on microbial diversity and respiration of red soil. Soil 47, 340–348. doi: 10.13758/j.cnki.tr.2015.02.023

[B4] Chen Z. G. Zhu Q. Wang W. H. Li J. (2006). Research on soil and water conservation effects of economic plant hedges with balanced fertilization in southern red and yellow soil areas. Soil Water Conserv. Res. 5, 248–251. doi: 10.1016/S1872-2032(06)60050-4

[B5] Deng Q. Cheng X. Hui D. Zhang Q. Li M. Zhang Q. (2016). Soil microbial community and its interaction with soil carbon and nitrogen dynamics following afforestation in central China. Sci. Total Environ. 541, 230–237. doi: 10.1016/j.scitotenv.2015.09.080 26410698

[B6] Doan T. T. Henry-des-Tureaux T. Rumpel C. Janeau J. L. Jouquet P. (2015). Impact of compost, vermicompost and biochar on soil fertility, maize yield and soil erosion in Northern Vietnam: a three year mesocosm experiment, Sci. Total Environ. 514, 147–154. doi: 10.1016/j.scitotenv.2015.02.005 25659313

[B7] Fidel R. Archontoulis S. Babcock B. Brown R. C. Dokoohaki H. Hayes D.. (2017). Commentary on ‘Current economic obstacles to biochar use in agriculture and climate change mitigation’regarding uncertainty, context-specificity and alternative value sources. Carbon Manage. 8, 215–217. doi: 10.1080/17583004.2017.1306408

[B8] Gao S. DeLuca T. H. (2018). Wood biochar impacts soil phosphorus dynamics and microbial communities in organically-managed croplands. Soil Biol. Biochem. 126, 144–150. doi: 10.1016/j.soilbio.2018.09.002

[B9] Ji R. L. Zhu Y. N. Tong X. W. Zhang A. L. Zhu B. F. Wang D. Q. (2008). Ammonia volatilization of bamboo- charcoal coated urea in soil and affecting factors. J. Guilin Univ. Technol. 28, 113–118.

[B10] Kim Y. C. Leveau J. McSpadden G. B. B. Pierson E. A. Pierson L. S. Ryu C. M. (2011). The multifactorial basis for plant health promotion by plant-associated bacteria. Appl. Environ. Microbiol. 77, 1548–1555. doi: 10.1128/AEM.01867-10 21216911 PMC3067257

[B11] Lehmann J. Rillig M. C. Thies J. Masiello C. A. Hockaday W. C. Crowley D. (2011). Biochar effects on soil biota–a review. Soil Biol. Biochem. 43, 1812–1836. doi: 10.1016/j.soilbio.2011.04.022

[B12] Li Y. B. Ba Y. L. Li S. Tian X. H. (2015). Combined addition of crop residues and their biochar increase soil organic C content and mineralization rate. J. Plant Nutr. Fertilizer 21, 943–950.

[B13] Liu Z. Y. (2005). Current situation and development advantages and potential of potato industry in Guizhou. Guizhou Agric. Sci. 3, 5–8.

[B14] Liu C. X. (2016). Preliminary isolation and characterization of antimicrobial substances of Lysobium chiliense X2-3. Shandong Agric. Univ. 1–62.

[B15] Lu H. Lashari M. S. Liu X. Ji H. Li L. Zheng J.. (2015). Changes in soil microbial community structure and enzyme activity with amendment of biochar-manure compost and pyroligneous solution in a saline soil from Central China. Eur. J. Soil Biol. 70, 67–76. doi: 10.1016/j.ejsobi.2015.07.005

[B16] Luo S. P. (2021). Nutrient stoichiometry and microbial community response to biochar addition in karst loess (Chongqing: Southwest University). doi: 10.27684/d.cnki.gxndx.2021.000107

[B17] Marta L. Ana A. Solange O. (2014). Legume growth-promoting rhizobia: An overview on the Mesorhizobium genus. Microbiological Res. 169, 2–17. doi: 10.1016/j.micres.2013.09.012 24157054

[B18] Minhas W. A. Hussain M. Mehboob N. Nawaz A. Ul-Allah S. Rizwan M. S.. (2020). Synergetic use of biochar and synthetic nitrogen and phosphorus fertilizers to improves maize productivity and nutrient retention in loamy soil, J. Plant Nutr. 43, 1356–1368. doi: 10.1080/01904167.2020.1729804

[B19] Mishra A. Kumar M. Bolan N. S. Kapley A. Kumar R. Singh L. (2021). Multidimensional approaches of biogas production and up-gradation: opportunities and challenges, Bioresour. Technol. 338, 125514. doi: 10.1016/j.biortech.2021.125514 34265593

[B20] Nannipieri P. Muccini L. Ciardi C. (1983). Microbial biomass and enzyme activities: production and persistence. Soil Biol. Biochem. 15, 679–685. doi: 10.1016/0038-0717(83)90032-9

[B21] Novak J. M. Ippolito J. A. Watts D. W. Sigua G. C. Ducey T. F. Johnson M. G. (2019). Biochar compost blends facilitate switchgrass growth in mine soils by reducing Cd and Zn bioavailability. Biochar 1, 97–114. doi: 10.1007/s42773-019-00004-7 35321098 PMC8939468

[B22] Partey S. T. Preziosi R. F. Robson G. D. (2014). Short-term interactive effects of biochar, green manure, and inorganic fertilizer on soil properties and agronomic characteristics of maize, Agric. Res. 3, 128–136. doi: 10.1007/s40003-014-0102-1

[B23] Prayogo C. Jones J. E. Baeyens J. Bending G. D. (2014). Impact of biochar on mineralisation of C and N from soil and willow litter and its relationship with microbial community biomass and structure. Biol. Fertility Soils 50, 695–702. doi: 10.1007/s00374-013-0884-5

[B24] Pyakurel A. Dahal B. R. Rijal S. (2019). Effect of molasses and organic fertilizer in soil fertility and yield of spinach in Khotang. Nepal Int. J. Appl. Sci. Biotechnol. 7, 49–53. doi: 10.3126/ijasbt.v7i1.23301

[B25] Shi Y. Liu X. Zhang Q. Gao P. Ren J. (2020). Biochar and organic fertilizer changed the ammonia-oxidizing bacteria and archaea community structure of saline–alkali soil in the North China Plain. J. Soils Sediments 20, 12–23. doi: 10.1007/s11368-019-02364-w

[B26] Sun M. Y. (2022). Investigating the mechanisms of ridge-furrow with plastic film mulching and fertilization on potato yields and greenhouse gas emissions in hilly area of the loess plateau (Hohhot, Inner Mongolia: Inner Mongolia Agricultural University).

[B27] Taschen E. Amenc L. Tournier E. Deleporte P. Malagoli P. Fustec J.. (2017). Cereal-legume intercropping modifies the dynamics of the active rhizospheric bacterial community. Rhizosphere 3, 191–195. doi: 10.1016/j.rhisph.2017.04.011

[B28] Uzoma K. C. Inoue M. Andry H. Fujimaki H. Zahoor A. Nishihara E. (2011). Effect of cow manure biochar on maize productivity under sandy soil condition. Soil Use Manag 27, 205–212. doi: 10.1111/j.1475-2743.2011.00340.x

[B29] Wang Z. (2022). Study on the mechanism of organic materials on structural changes, water movement and ecological functions of alkalized soil. Ningxia Univ. 1–160.

[B30] Wang L. H. Guo X. D. Tan X. L. Guo T. W. (2016). Effects of different crop rotations on enzyme activities and microbial quantities in potato soil. Agric. Res. Arid Areas 34, 109–113.

[B31] Wang Z. Y. Wang Y. X. Chen Z. R. (1991). The nature of soybean-soybean cropping. Soybean Sci. 10, 31–36.

[B32] Wang X. M. Yan B. G. Zhao G. Zhao J. X. Shi L. T. Liu G. C. (2018). Responses of Dodonaea viscosa growth and soil biological properties to nitrogen and phosphorus additions in Yuanmou dry-hot valley. J. Mt. Science. 15, 1283–1298. doi: 10.1007/s11629-017-4544-3

[B33] Warnock D. D. Lehmann J. Kuyper T. W. Rillig M. C. (2007). Mycorrhizal responses to biochar in soil–concepts and mechanisms. Plant soil. 300, 9–20.

[B34] Wu F. Wang X. (2006). Effect of monocropping and rotation on soil microbial community diversity and cucumber yield and quality under protected cultivation. XXVII Int. Hortic. Congress-IHC2006: Int. Symposium Adv. Environ. Control Automation. 761, 555–561. doi: 10.17660/actahortic.2007.761.77

[B35] Xiao X. Gui Y. F. Zhu Y. Fu J. Zhu X. S. Zhu X.. (2021). Effect of microbial inoculations on structure and activity of paddy soil bacterial community. Southwest China J. Agric. Sci. 34, 2174–2181. doi: 10.16213/j.cnki.scjas.2021.10.016

[B36] Yang H. Hu J. W. Huang X. F. Fan M. Y. Li J. L. (2015). Research on soil fertility of golden prickly pear cultivation base in karst region. Soil Water Conserv. Res. 22, 50–55. doi: 10.13869/j.cnki.rswc.2015.03.010

[B37] Ye L. Camps-Arbestain M. Shen Q. Lehmann J. Singh B. Sabir M.. (2020). Biochar effects on crop yields with and without fertilizer: a meta-analysis of field studies using separate controls. Soil Use Manag 36, 2–18. doi: 10.1111/sum.12546

[B38] Ye Z. X. Zhang L. M. Huang Q. Y. Tan Z. X. (2019). Development of a carbon-based slow release fertilizer treated by bio-oil coating and study on its feedback effect on farmland application, J. Clean. Prod. 239, 118085. doi: 10.1016/j.jclepro.2019.118085

[B39] Yu L. Lu X. He Y. Brookes P. C. Liao H. Xu J. M. (2016). Combined biochar and nitrogen fertilizer reduces soil acidity and promotes nutrient use efficiency by soybean crop, J. Soils Sediments. 17, 599–610. doi: 10.1007/s11368-016-1447-9

[B40] Zhai L. M. CaiJi Z. M. Liu J. Wang H. Y. Ren T. Z. Gai X. P.. (2015). Short-term effects of maize residue biochar on phosphorus availability in two soils with different phosphorus sorption capacities, Biol. Fertil. Soils. 51, 113–122. doi: 10.1007/s00374-014-0954-3

[B41] Zhang L. C. He C. F. Zhang W. M. Zhang C. H. Qiu H. Z. (2017). Effects of fertilizer treatments on soil organic matter content and microbial flora of potato. J. Gansu Agric. Univ. 52, 27–33. doi: 10.13432/j.cnki.jgsau.2017.02.005

[B42] Zhang X. Huang G. Bian X. Jiang X. Zhao Q. (2012). Effects of intercropping on quality and yield of maize grain, microorganism quantity, and enzyme activities in soils. Shengtai Xuebao/Acta Ecologica Sin. 32, 7082–7090. doi: 10.5846/stxb201110151526

[B43] Zhang M. Li Z. Haggblom M. M. Young L. He Z. Li F.. (2020). Characterization of nitrate-dependent As (III)-oxidizing communities in arsenic-contaminated soil and investigation of their metabolic potentials by the combination of DNA-stable isotope probing and metagenomics. Environ. Sci. Technology. 54, 7366–7377. doi: 10.1021/acs.est.0c01601 32436703

[B44] Zhang Y. Tan Q. Hu C. Zheng C. Gui H. Zeng W.. (2015). Differences in responses of soil microbial properties and trifoliate orange seedling to biochar derived from three feedstocks. J. Soils Sediments 15, 541–551. doi: 10.1007/s11368-014-1032-z

[B45] Zhang X. Wang D. Jiang C. C. Zhu P. Lei J. Peng S. A. (2013). Effect of biochar on physicochemical properties of red and yellow brown soils in the South China Region. Chin. J. Eco-Agriculture 21, 979–984. doi: 10.3724/SP.J.1011.2013.00979

[B46] Zhang M. Xu Y. K. Ma Z. X. Zhang R. H. Gou J. L. Chen L.. (2017). Effect of top-dressing fertilizer with fertilizer applicator on yield, tuber traits of spring potato and soil fertility in guizhou province. J. Irrigation Drainage 36, 35–40. doi: 10.13522/j.cnki.ggps.2017.09.007

[B47] Zhao Z. H. Zhang C. Z. Cai T. Y. Liu C. H. Zhang J. B. (2015). Effects of different stable organic matters on physicochemical properties of lime concretion black soil and maize yield. Chin. J. Eco-Agriculture 23, 1228–1235. doi: 10.13930/j.cnki.cjea.150546

