# Supplementary material for: Increase in potato yield by the combined application of biochar and organic fertilizer: key role of rhizosphere microbial diversity
Source: Front Plant Sci. 2024 May 15;15:1389864. doi: 10.3389/fpls.2024.1389864 (PMC11133632; doi:10.3389/fpls.2024.1389864)
Supplement: Supplementary file 1 [file DataSheet_1.docx]

Fig. S1. Community structural characteristics (A) and dominant bacteria (B) of rhizosphere soil at the bacteriophyta level under different mix proportions of biochar and organic fertilizer. Different lowercase letters in the column indicate significant differences among different treatments (*p*<0.05). Pro: *Proteobacteria*; Act: *Actino- bacteria*; Gem: *Gemmatimonadetes*; Chl: *Chloroflexi*; Bac: *Bacteroidetes*; Aci: *Acidobacteria*; Pat: *Patescibacter- ia*; Fir: *Firmicutes*; Dei: *Deinococcus-Thermus*; Pla: *Planctomycetes*.


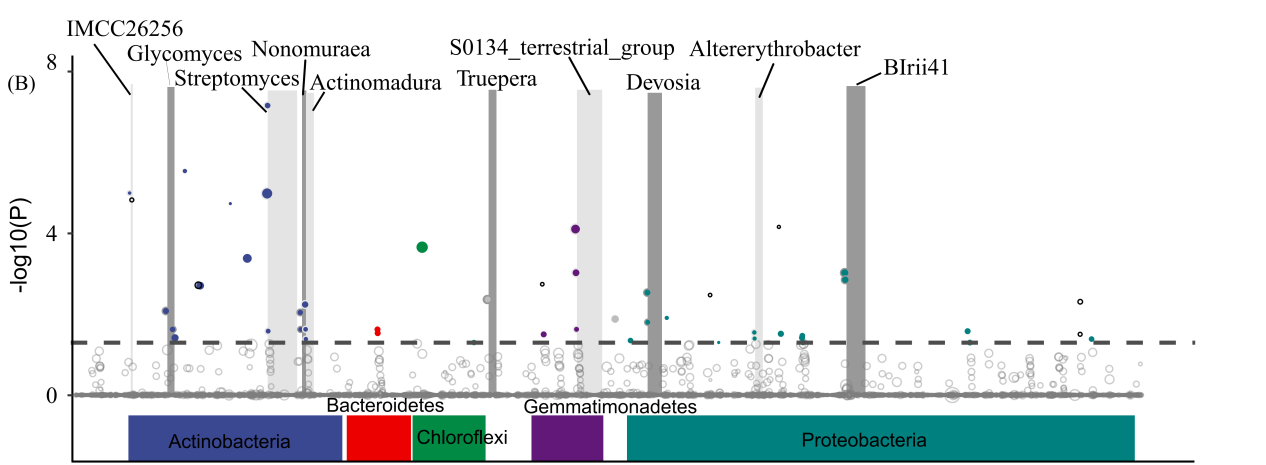

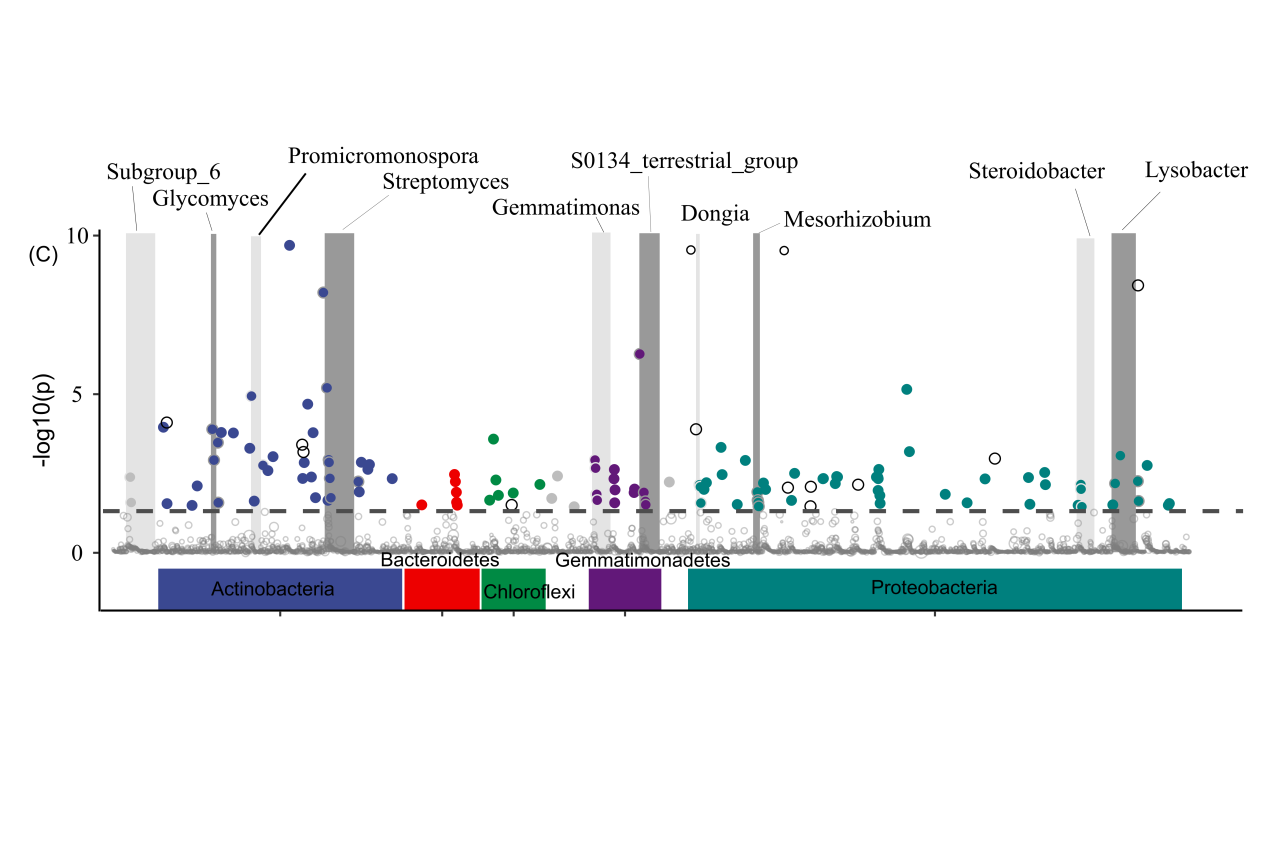

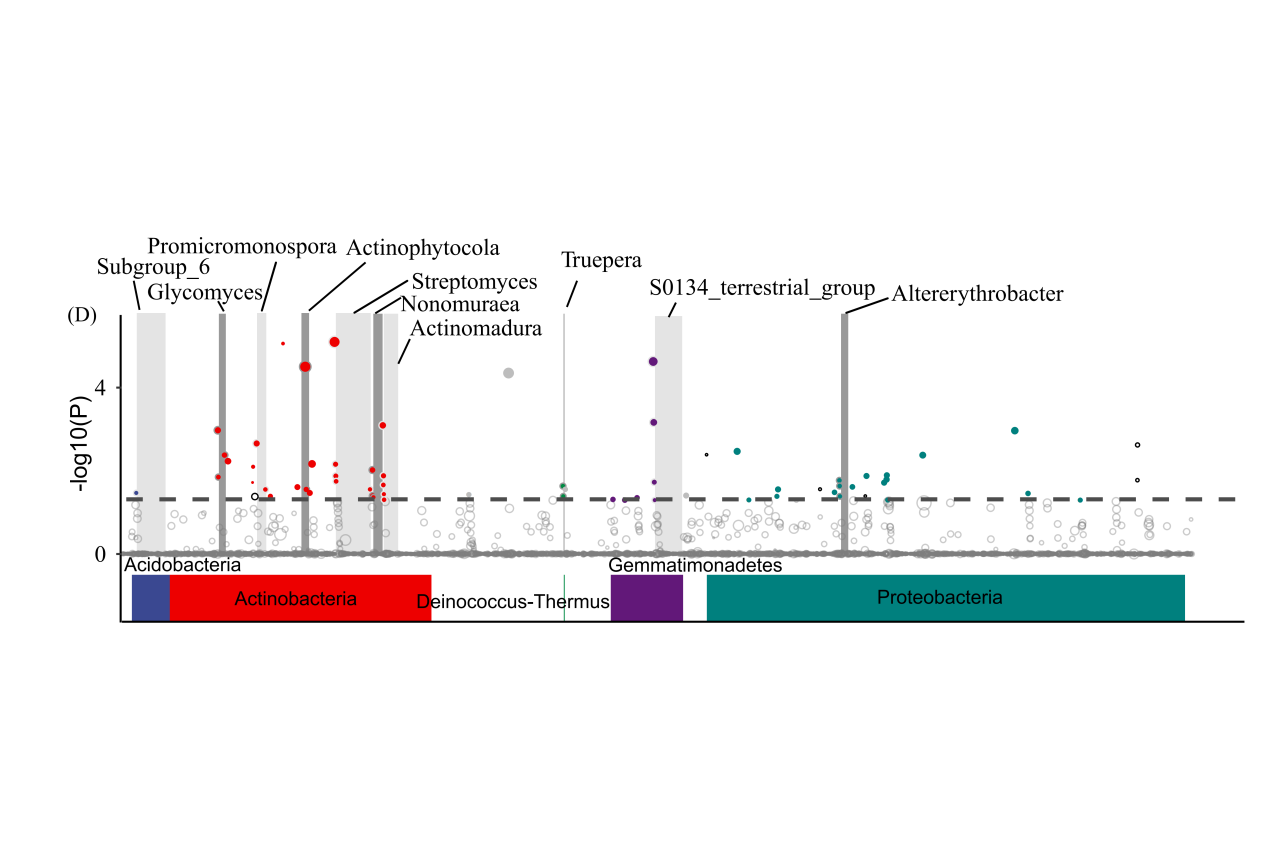

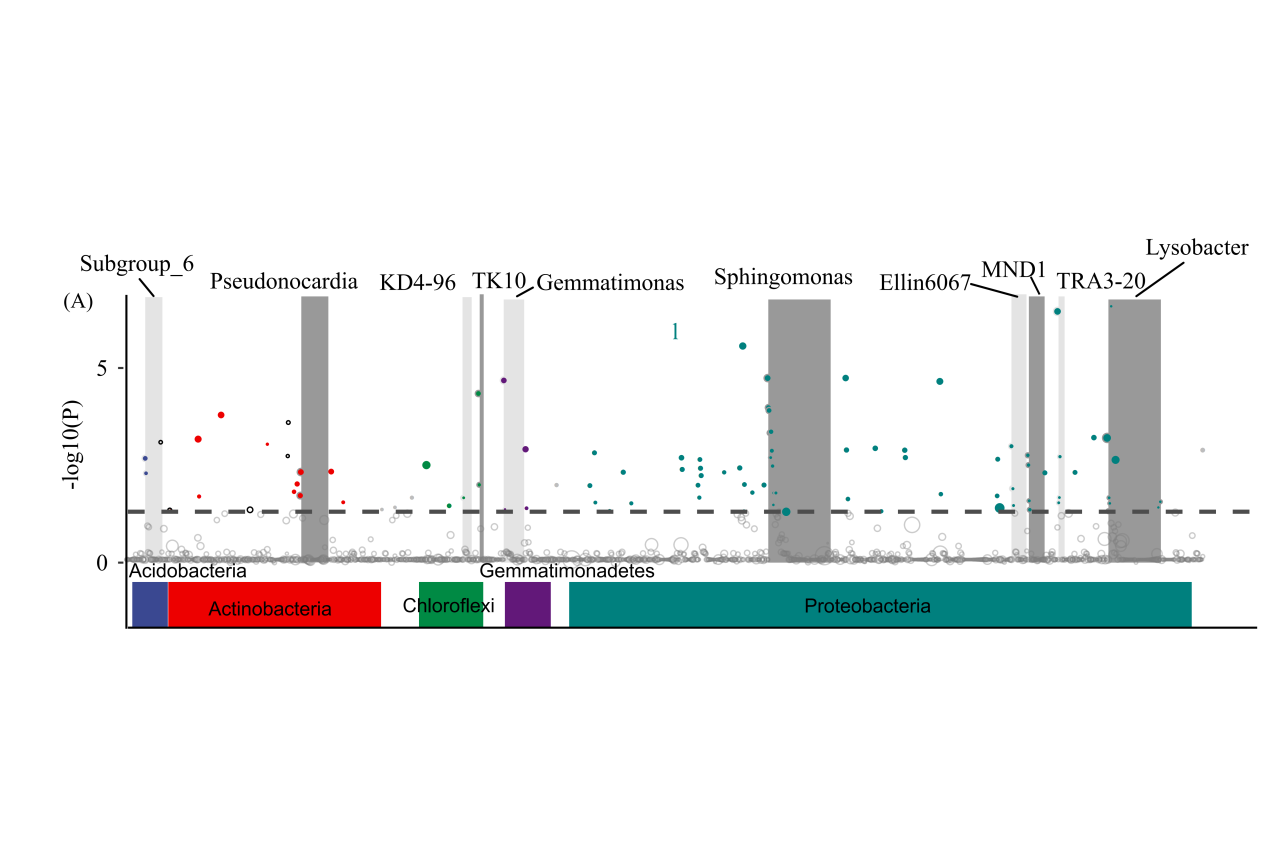


Fig. S2. MetagenomeSeq analysis of rhizosphere bacterial species differences and marker species. ASVs enriched in the B:O=1:0 (A), B:O=1:1 (B), B:O=1:2 (C), and B:O=1:3 treatments (D) with respect to CK.


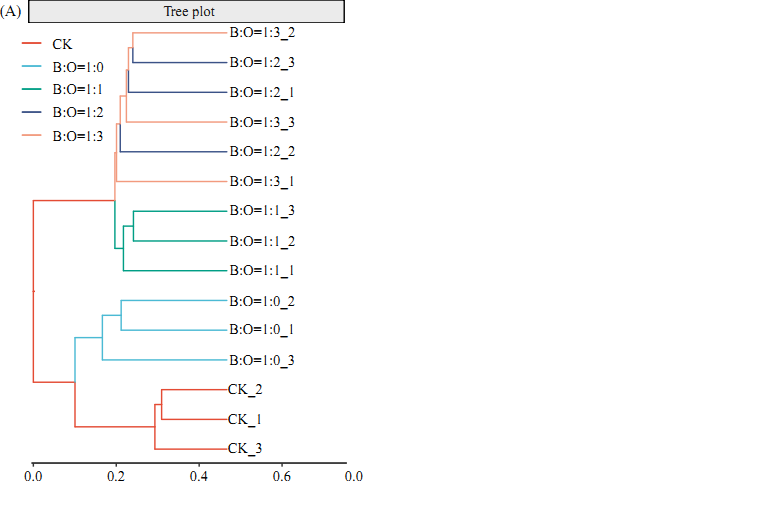

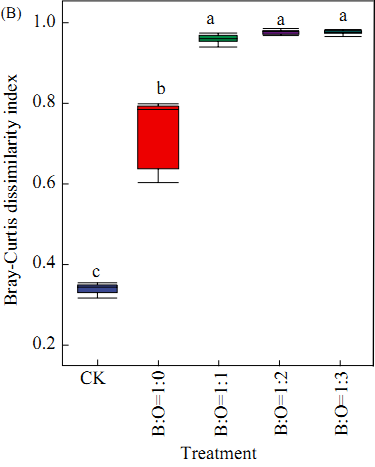


Fig. S3. Hierarchical clustering analysis (A) and intergroup difference analysis (B) of rhizosphere bacterial communities.


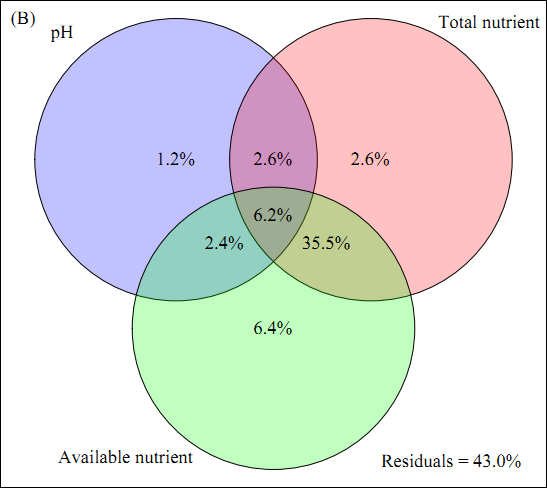

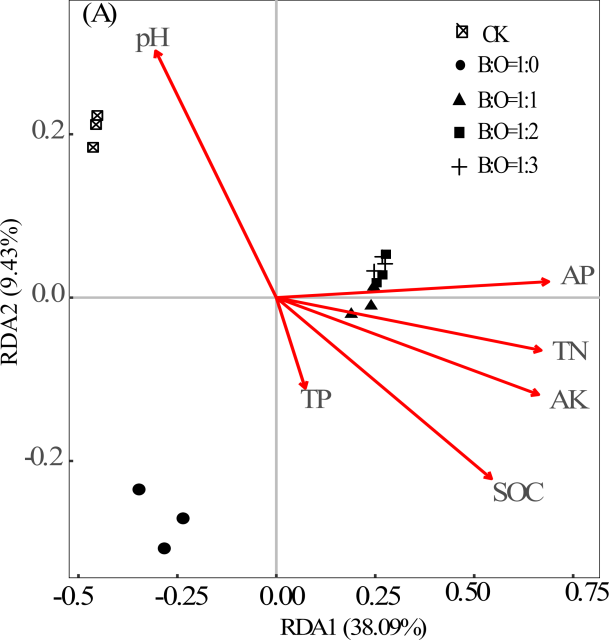


Fig. S4. RDA (A) and variance decomposition analysis (B) of the relationship between rhizosphere soil properties and bacterial communities.


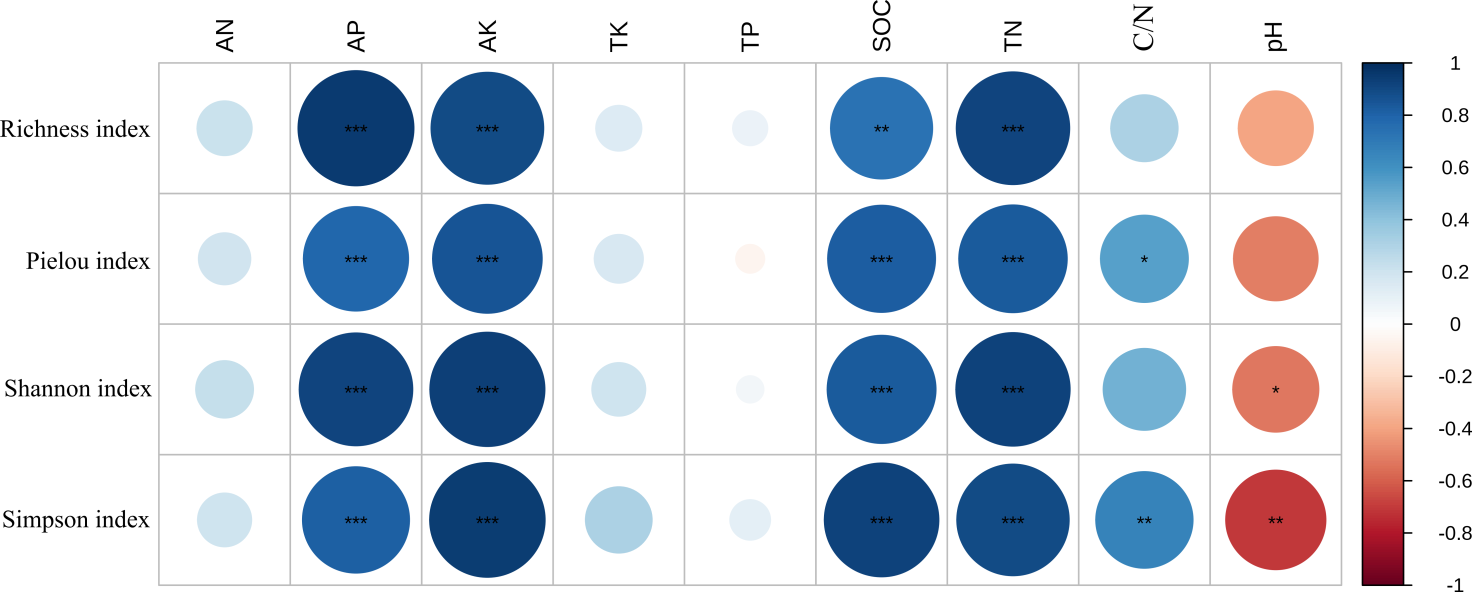


Fig. S5. Correlation analysis between rhizosphere soil properties and bacterial diversity index. **p*<0.05; ***p*<0.01; *** *p*<0.001.
